# Habenular expression of rare missense variants of the β4 nicotinic receptor subunit alters nicotine consumption

**DOI:** 10.3389/fnhum.2014.00012

**Published:** 2014-01-27

**Authors:** Marta A. Ślimak, Jessica L. Ables, Silke Frahm, Beatriz Antolin-Fontes, Julio Santos-Torres, Milena Moretti, Cecilia Gotti, Inés Ibañez-Tallon

**Affiliations:** ^1^Molecular Neurobiology Group, Max Delbrück Center for Molecular MedicineBerlin, Germany; ^2^Laboratory of Molecular Biology, The Rockefeller UniversityNew York, NY, USA; ^3^Consiglio Nazionale delle Ricerche Institute of Neuroscience and Biometra Department, University of MilanMilan, Italy

**Keywords:** medial habenula, nicotine consumption, SNP, lentivirus transduction, electrophysiological recordings, smoking dependence

## Abstract

The *CHRNA5-CHRNA3-CHRNB4* gene cluster, encoding the α5, α3, and β4 nicotinic acetylcholine receptor (nAChR) subunits, has been linked to nicotine dependence. The habenulo-interpeduncular (Hb-IPN) tract is particularly enriched in α3β4 nAChRs. We recently showed that modulation of these receptors in the medial habenula (MHb) in mice altered nicotine consumption. Given that β4 is rate-limiting for receptor activity and that single nucleotide polymorphisms (SNPs) in *CHRNB4* have been linked to altered risk of nicotine dependence in humans, we were interested in determining the contribution of allelic variants of β4 to nicotine receptor activity in the MHb. We screened for missense SNPs that had allele frequencies >0.0005 and introduced the corresponding substitutions in *Chrnb4*. Fourteen variants were analyzed by co-expression with α3. We found that β4A90I and β4T374I variants, previously shown to associate with reduced risk of smoking, and an additional variant β4D447Y, significantly increased nicotine-evoked current amplitudes, while β4R348C, the mutation most frequently encountered in sporadic amyotrophic lateral sclerosis (sALS), showed reduced nicotine currents. We employed lentiviruses to express β4 or β4 variants in the MHb. Immunoprecipitation studies confirmed that β4 lentiviral-mediated expression leads to specific upregulation of α3β4 but not β2 nAChRs in the Mhb. Mice injected with the β4-containing virus showed pronounced aversion to nicotine as previously observed in transgenic Tabac mice overexpressing *Chrnb4* at endogenous sites including the MHb. Habenular expression of the β4 gain-of-function allele T374I also resulted in strong aversion, while transduction with the β4 loss-of function allele R348C failed to induce nicotine aversion. Altogether, these data confirm the critical role of habenular β4 in nicotine consumption, and identify specific SNPs in *CHRNB4* that modify nicotine-elicited currents and alter nicotine consumption in mice.

## Introduction

As the leading preventable cause of cancer and death, nicotine use and dependence has been the subject of a multitude of genetic studies in the past decade. By far, the strongest and most replicable evidence exists for a role of the *CHRNA5-A3-B4* gene cluster on chromosome 15q25 in nicotine addiction, which encodes three subunits of nicotinic acetylcholine receptors (nAChR). Two single nucleotide polymorphisms (SNPs) in particular, rs16969968 in *CHRNA5* and rs1051730 in *CHRNA3*, have been linked to smoking-related behaviors in multiple studies (Bierut et al., 2007; Saccone et al., 2007; Thorgeirsson et al., 2008). Though not as strongly linked, the β4 subunit has been nominally associated with smoking quantity in several studies (Saccone et al., 2009; Harari et al., 2012). Variants located upstream of *CHRNB4* affect the age at which individuals transition to daily and habitual smoking (Kapoor et al., 2012), while another study found that SNP rs12914008 in *CHRNB4* is associated with less abstinence over time (Sarginson et al., 2011). Rare missense variants in *CHRNB4* (T375I and T91I) and in *CHRNA3* (R37H) are associated with lower risk for nicotine dependence and fewer cigarettes per day (Haller et al., 2012).

Despite the preponderance of evidence linking the *CHRNA5-A3-B4* gene cluster to nicotine-related diseases, very little evidence exists as to how mutations in these receptors alter their function. Cumulative data from rodent models suggest that *CHRNA5* and *CHRNB4*, particularly in the medial habenula (MHb)-interpeduncular (IPN) circuit, mediate the aversive properties of nicotine (Salas et al., 2009; Fowler et al., 2011; Frahm et al., 2011) and withdrawal (Salas et al., 2009; Gorlich et al., 2013). For example, animals lacking the α5 nAChR subunit self-administer more nicotine (Fowler et al., 2011) and do not display somatic signs of nicotine withdrawal (Salas et al., 2009). Restoration of *CHRNA5* to the MHb leads to normalization of nicotine consumption, i.e., the mice reach a plateau in administration, suggesting that it is the aversive aspect that limits intake (Fowler et al., 2011). Overexpression of the entire human gene cluster in mice leads to increased sensitivity to nicotine with higher activation of the MHb and reduced activation of the VTA (Gallego et al., 2012). We previously demonstrated that overexpression of the β4 nAChR subunit in the Tabac transgenic mouse model is sufficient to increase sensitivity to the aversive properties of nicotine and decrease consumption. Overexpression of β4 also leads to increased surface expression of α3, as β4 is the rate-limiting subunit for assembly (Frahm et al., 2011). In particular we previously showed that β4 is rate-limiting for α3β4 activity since when overexpressed it increases the density of α3β4 receptors at the plasma membrane, as well as potentiates α3β4 currents *in vitro* and in a transgenic mouse model (Frahm et al., 2011). The ability of β4 to enhance nicotine-evoked currents depends on a unique single residue S435, which does not exist in other βnAChR subunits and can confer this capability to β2 when introduced in the corresponding arginine residue (Frahm et al., 2011).

This critical residue (S435) in β4 is located within the membrane-associated stretch in the intracellular vestibule of the receptor (Frahm et al., 2011). Sequence alignments identified other SNPs mapping to the intracellular vestibule, one of them being the most common SNP associated with nicotine use, rs16969968, leading to an amino acid substitution (D398N) in the α5 subunit. Functional analysis of this SNP in α5 demonstrated that mutation of this residue within the highly electrostatically charged intracellular vestibule of the nAChR significantly reduces nicotine-evoked currents. Behaviorally, this genetic variant had profound effects: Tabac mice with strong aversion to nicotine due to increased expression of β4, reverted to nicotine preference upon viral-mediated expression of the α5 D398N variant (Frahm et al., 2011). Given that several SNPs in β4 have been identified in humans, particularly in the intracellular vestibular region, we were interested in determining their contribution to α3β4 receptor function. Based on reported validation studies and heterozygosity rates, we selected 14 out of 67 missense SNPs identified in the coding region of *CHRNB4*. Expression of these missense β4 variants in oocytes indicated that amino acid substitutions corresponding to 12 of these 14 SNPs led to changes in the electrophysiological properties of the channel (Liang et al., 2005; Haller et al., 2012). We further analyzed four of these SNPS in hippocampal neurons, and found that the two variants most associated with decreased risk of nicotine dependence, A90I and T374I (Haller et al., 2012), and a third variant D447Y, augmented nicotine-mediated α3β4 nAChR currents, while the fourth analyzed SNP, previously associated with sporadic amyotrophic lateral sclerosis (sALS) (Moriconi et al., 2011), reduced nicotine currents. Mice injected in the MHb with lentiviruses carrying the wild-type β4 subunit or β4 rare missense variants, showed aversion or preference to nicotine, depending on whether the variant increased or decreased nicotine currents. These results indicate that nicotine consumption is regulated by β4^*^ nAChR activity in the MHb.

## Materials and methods

### Animals

Transgenic Tabac mice were obtained from GENSAT (Gong et al., 2003) and backcrossed to a C57BL/6 background. All transgenic animals used for experiments were hemizygous. C57BL/6 male mice were purchased from Charles River (Germany) and allowed to habituate to the facility for 1 week before use in lentiviral experiments. Mice were housed with *ad libitum* access to food and water in a room air conditioned at 22–23°C with a standard 12 h light/dark cycle, with a maximum of five animals per cage. All procedures were in accordance with ethical guidelines laid down by the local governing body and approved by the Landesant Für Gesundheit und Soziales, Berlin and the Institutional animal use and care committee IACUC, New York).

### Two-electrode voltage-clamp recordings of xenopus laevis oocytes

**Figure 1:**
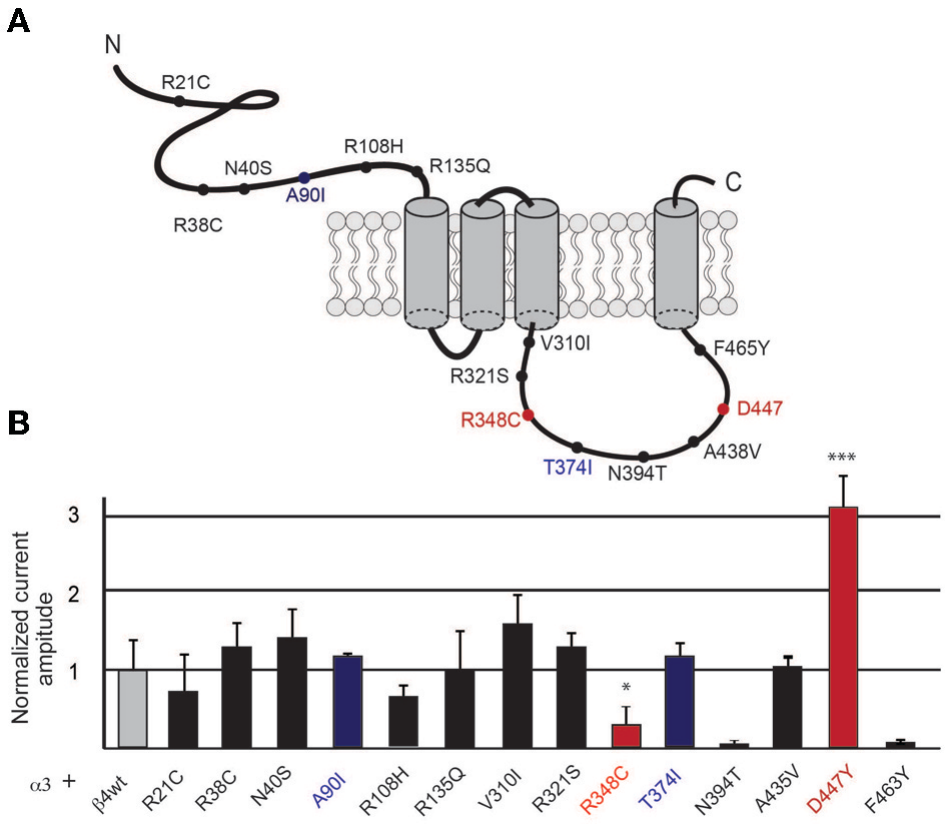
**Selected β4 missense variants and nicotine-evoked currents in oocytes. (A)** Graphic representation of the β4 nAChR subunit indicating the location of the rare missense variants at the extracellular and intracellular loops of the receptor subunit. **(B)** Electrophysiological macroscopic recordings in *Xenopus laevis* oocytes injected with equal amounts of the wild type transcripts for α3 and β4 (1 ng α3 + 1 ng β4) or with equal amounts of transcripts for wild type α3 and each of the indicated β4 variants (*n* = 10 oocytes per condition). The residues in blue indicate the mouse variants corresponding to the two human β4 SNPs shown to have increased nicotine currents. Red indicates two additional residues that significantly differ from the wild type β4 nAChR subunit in gray. All values are expressed as mean ± s.e.m. One-way ANOVA *F*_(14, 91)_ = 9.1, *p* < 0.0001; Dunnett's Multiple Comparison *post-hoc* ^*^p < 0.05, ^***^p < 0.001.

**Figure 2:**
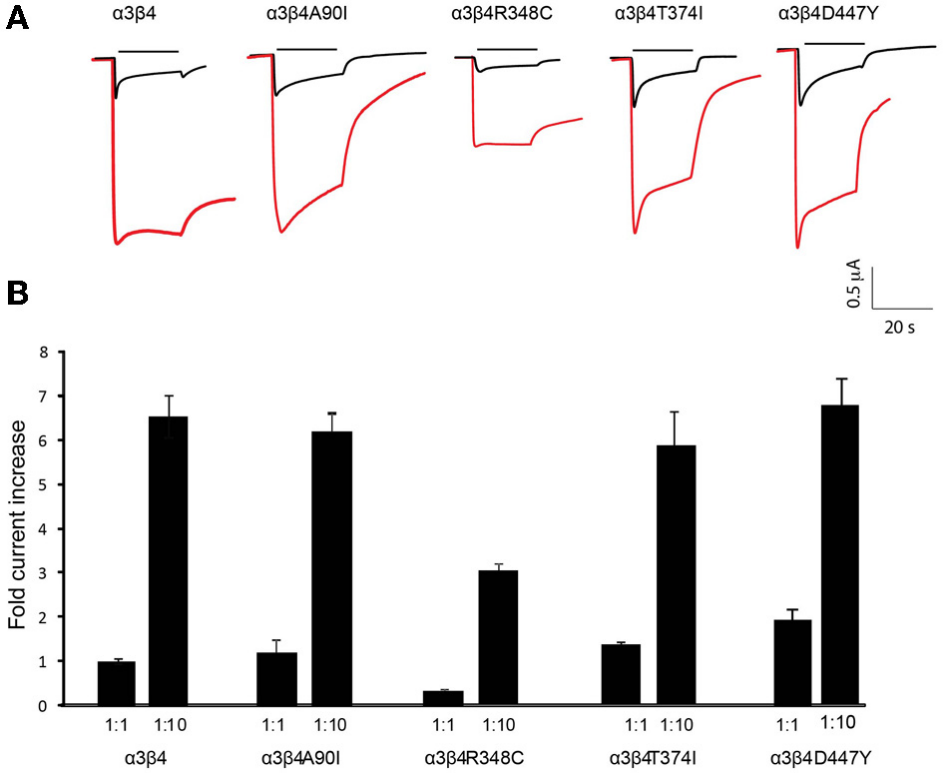
**Increased currents by overexpression of β4 variants. (A)** Representative traces of two-electrode-voltage-clamp recordings in oocytes injected with either equal amounts of α3 and β4 transcripts (1 ng α3 + 1 ng β4, 1:1 ratio; black traces) or with 10 times more of the β4 transcript (1 ng α3 + 10 ng β4, 1:10 ratio; red traces). Horizontal line above each trace indicates nicotine (1 mM) application. **(B)** Bar graph indicates the fold increase of the current amplitude elicited by 1 mM nicotine with respect to a3b4 wt oocytes injected at 1:1 (*n* = 10 oocytes per condition) for oocytes injected with 1:1 or 1:10 rations of the indicated β4 variants A90I, T374I, D447Y, and R348C. All values are expressed as mean ± s.e.m.

cDNA encoding for the mouse α3 and β4 nAChR subunit was subcloned into the pCS2A plasmid for oocyte expression. Site-directed mutations corresponding to the selected SNPs were introduced in the β4 nAChR subunit using QuikChange Site-Directed mutagenesis kit (Stratagene, catalog # 200519). RNA transcripts were prepared using the mMESSAGE mMACHINE kit (Ambion, catalog # AM1344M) as described (Ibanez-Tallon et al., 2004). Oocytes were surgically extracted and prepared as described (Sturzebecher et al., 2010). For electrophysiological recordings of the 14 β4 variants shown in Figure 1, each oocyte was injected with 20 nl of a cRNA mix containing 1 ng of α3 + 1 ng of β4 (β4 wt or each of the 14 indicated β4 variants). For electrophysiological recordings of the β4 variants A90I, T374I, D447Y, and R348C shown in Figure 2, each oocyte was injected at 1:1 and 1:10 ratios, with either 1 ng of α3 + 1 ng of β4 (wt or variants): or 1 ng of α3 + 10 ng of β4 (wt or variants). Macroscopic currents were recorded 4 days after injection with a GeneClamp 500 B amplifier (Axon Instruments) using a two-electrode voltage clamp with active ground configuration as described in Frahm et al. (2011). Nicotine tartrate salt (Sigma-Aldrich, catalog # N5260) was applied at the concentration of 1mM. Mean fold current increase was evaluated by dividing peak amplitudes of 5–10 single oocytes expressing receptors containing each of the polymorphic variants of β4 nAChR by peak amplitudes of the ones carrying native β4 nAChR at 1:1 and 1:10 ratio.

### Lentivirus production

Recombinant lentiviral vectors were prepared using transient transfection of HEK293T cells as described in (Auer et al., 2010).

### Whole-cell patch clamp recordings in primary hippocampal neurons

Dissociated hippocampal cultures were prepared from embryonic day 19 rat embryos and prepared as described in (Auer et al., 2010). After 9 days in culture, neurons were infected with lentiviruses carrying the α3 and one of the variants of β4 nAChR subunit. Three to four days after lentivirus infection, nicotine-elicited currents were analyzed by whole-cell patch-clamp recordings. The internal pipette solution contained (mM): 130 KCl, 2 MgCl_2_, 0.5 CaCl_2_, 5 EGTA, 10 HEPES, pH 7.3, osmolarity 280 (resistance 5–7 MΩ). 30 μM nicotine tartrate was locally applied (50 ms, 18–10 psi) with a pressure device (PR-10, ALA Scientific Instruments) connected to a focal perfusion system (VM4, ALA Scientific Instruments) controlled with a trigger interface (TIB 14S, HEKA). The pipette was moved within 15–20 μm of the recorded cell with a motorized micromanipulator (LN mini 25, control system SM-5, Luigs and Neumann) for drug application and retracted after the end of the puff to minimize desensitization. Currents were recorded with a HEKA amplifier (EPC 10) using PatchMaster software (V2.20, HEKA), and analyzed with FitMaster software (V2.3, HEKA). Membrane potential was held at −70 mV.

### Stereotactic viral injections in the MHb

Stereotactic injections were performed as described in Frahm et al. (2011). Briefly, 1 μl of concentrated lentivirus (2–5 × 10^8^ TU/ml) was injected bilaterally into the MHb of 8 week-old C57Bl/6 male mice at each of the following coordinates from bregma (Franklin and Paxinos, 2008): antero-posterior −1.40 and −1.74, lateral ±0.33, dorso-ventral (from skull level) −2.7 and −2.74. Behavioral experiments started 2 weeks after injection with either the wild type β4-carrying lentivirus pFU-β4-IRES-EGFP or virus carrying one of the β4-point mutants. Expression of eGFP using the same pFU lentiviral vectors has been shown after just 1 week (Dittgen et al., 2004). Immunoprecipitation assays or sacrifice followed by transcardial perfusion with 4% paraformaldehyde in PBS were done 4 weeks post-injection. Verification of the injection site for each animal in the behavioral assays was performed on 40 μm coronal brain sections immunostained with rabbit polyclonal anti-GFP antibody (Invitrogen, catalog # A11122) diluted 1:1000 as previously described (Frahm et al., 2011; Gorlich et al., 2013). Animals with lentiviral injection placement outside the MHb were excluded from behavioral analysis, leaving 6–8 animals per group.

### Immunoprecipitation of [^125^I] epibatidine-labeled receptors by subunit-specific antibodies

Four weeks post-injection, habenula were dissected from C57BL/6 mice injected with either the β4-carrying lentivirus pFU-β4-IRES-EGFP or control virus pFU–PE, and were processed as previously described (Grady et al., 2009). Briefly, 2% Triton X-100 habenula extracts were preincubated with 2 μM αBgtx (Tocris, Bristol, UK catalog # 2133), labeled with 0.5 nM [**^125^I**] Epibatidine (specific activity 2200 Ci/mmol, Perkin Elmer catalog #NEX358) and incubated overnight with a saturating concentration of affinity-purified anti-subunit α3, β4, and β2 specific polyclonal IgG produced and characterized by Grady et al. (2009). These polyclonal antibodies [supplementary Table of Grady et al. (2009)] were produced against the following peptide sequences TRPTSTEEDAPKTRNFYGAELSNLNC in the cytosolic loop of mouse α3, VSSHTAGLPRDARLRSSGRFREDLQEALEG in the cytosolic loop of rat β4, and RQREREGAGALFFREAPGADSCTY in the cytosolic loop of human β2. As described in (Grady et al., 2009), the specificity of the anti β4 and anti β2 antibodies, was tested by immunoprecipitation using ^3^H- or ^125^I-Epibatidine labeled receptors obtained from tissues of wt and β4 and β2 KO mice respectively; specific immunopreciptation was obtained in wt but not in the respective KO mice. The anti-α3 antibody specificity was determined by specific immunoprecipitation of anti-α3 antibodies only in HEK cells transfected with the α3β4 or α3β2 subtypes and not in HEK cells transfected with the human α2β2, α2β4, α4β2, and α4β4 subtypes. After incubating with the specific polyclonal antibodies, the immunoprecipitated fractions from habenula extracts were recovered by incubating the samples with beads containing bound anti-rabbit goat IgG (Technogenetics). The level of antibody immunoprecipitation was expressed as femtomoles of immunoprecipitated receptors per milligram of protein.

### Two-bottle nicotine consumption

The two-bottle drinking test was used to measure the consumption to nicotine relative to water, as described in Meliska et al. (1995), Robinson et al. (1996), Butt et al. (2005), Glatt et al. (2009). We used a moderate dose of 50 μg/ml nicotine (Matta et al., 2007) since at this dose we previously observed a large difference in nicotine consumption between wild type and Tabac mice (Frahm et al., 2011). Adult male Tabac mice or lentiviral injected C57BL/6 mice were single-housed and tested in their home cages. For the first 3 days, nicotine naïve mice were presented with two bottles of 2% saccharin in tap water for acclimation to the test conditions. After 3 days, the volume consumed from each bottle was recorded to determine any baseline side preference. The difference in volume before and after 3 day consumption was measured by weighting the cage bottles (0.5 ml is the smallest difference that can be measured by weighting). After the acclimation period, the solution in one of the bottles was replaced with nicotine tartrate (50 μg/ml) in 2% saccharin in tap water, and the volume drunk from each bottle was measured after 3 days. To account for side-bias, nicotine was presented on the side that was less preferred. Bottles were equipped with sipper tubes with ball bearings to prevent volume loss by leakage. Percent nicotine consumption was expressed as a ratio of the volume of nicotine solution consumed divided by the total fluid intake (ml nicotine/ml total × 100).

### Statistical analysis

All data are presented as the mean ± s.e.m. For electrophysiological and behavioral tests, ANOVA followed by Dunnett's multiple comparisons *post-hoc* was used to analyze the data (GraphPad Prism5). The number of oocytes, neurons, or animals per group is indicated in the figure legend. The statistical values (F, degrees of freedom) can be found in the results section. Student's *t*-test was used to analyze the immunoprecipitation data

## Results

### Selection of missense SNPS in *CHRNB4* for functional analysis

We searched the SNP database for missense polymorphisms in the coding region (cSNPs) of the *CHRNB4* gene (http://www.ncbi.nlm.nih.gov/projects/SNP/snp_ref.cgi?geneId=1143). The database reports 67 missense variants with very low or not determined minor allele frequency (MAF). Given that validation studies have been reported for only a third of these missense variants, and that informative SNPs need to be polymorphic on the two different alleles (Li et al., 2007), we selected 14 variants that had either an heterozygosity index *p* ≥ 0.004, or were validated by frequency or genotype data (minor alleles observed in at least two chromosomes) (Table 1). We introduced the corresponding mutations in the mouse *Chrnb4* gene by site-directed mutagenesis of the indicated nucleotides in the mouse sequence (Table 1). It is interesting to note that 8 of the 14 selected SNPs result in a change in the charge or hydrophobicity of the aminoacid residue and that 6 of these SNPs correspond to mutations of arginine (R) residues (positively charged) (Table 1). Given that all of these variants are in the extracellular domain and intracellular loop of the β4 subunit (Figure 1A), this suggests that some of these variants may alter the hydrophobicity or electrostatic charge of these functional domains in the β4 subunit.

**Table 1:** **Missense SNPs in *CHRNB4* and corresponding variants introduced in the mouse sequence**.

**rs number**	**MAF**	**HET**	**^h^Missense**	**^m^Missense**	**^m^Mut**	**Aa charge**	**SIFT**
71653605	ND	0.039	R22C	R21C	**TGC**	+ to O	TOLERATED
72648898	ND	0.004	R39C	R38C	**TGC**	+ to O	DAMAGING
75495090	0.0032	0.012	N41S	N40S	**AGC**	O to O	DAMAGING
12914008	0.0165	0.062	T91I	A90I	**ATC**	H to H	DAMAGING
149832833	0.0005	0.001	R109H	R108H	**CAC**	+ to +	TOLERATED
56095004	0.0037	0.012	R136Q	R135Q	**CAG**	+ to O	TOLERATED
143402850	ND	0.001	V311I	V310I	**ATC**	H to H	TOLERATED
79914661	ND	0.02	R322S	R321S	**AGT**	+ to O	DAMAGING
56235003	0.0051	0.014	R349C	R348C	**TGC**	+ to O	DAMAGING
61737499	0.0005	0.016	T375I	T374I	**ATC**	O to H	TOLERATED
75124790	ND	0.129	N391T	N394T	**ACC**	O to O	TOLERATED
56317523	0.0018	0.004	A435V	A348V	**GTA**	H to H	DAMAGING
56258098	ND	0.005	D444Y	D447Y	**TAT**	− to O	TOLERATED
79647370	ND	0.002	F462Y	F465Y	**TAC**	H to O	TOLERATED

Reference ID numbers (rs number), validated minor allele frequencies (MAF) and heterozygosity rates (HET) of the SNPs in the coding sequence of CHRNB4 that cause non-synonymous missense mutations in human carriers (

h missense). The corresponding missense variants were introduced in the mouse sequence (

m missense) with the indicated nucleotide substitutions (

m mut) in bold. In most cases the missense variant changed the charge of the aminoacid (Aa) (indicated as +, positively charged; O, polar no charge; − negatively charged; H, hydrophobic). The last column indicates the SIFT (Sorting Intolerant from Tolerant) value predicts the impact of a residue substitution on protein function (http://siftdna.org/www/Extended_SIFT_chr_coords_submit.html).

### Nicotine-elicited currents of α3β4 nAChR and missense β4 variants

To begin to assess the influence of the selected missense β4 variants on nicotine-mediated currents, we performed two electrode voltage clamp recordings in *Xenopus laevis* oocytes. Oocytes were co-injected with equal amounts of transcripts encoding the α3 nAChR subunit and wild-type (wt) β4 or α3 and each of the β4 point mutants (1 ng of α and 1 ng of βper oocyte). As shown in Figure 1, nicotine-elicited currents were observed for all the selected missense β4 variants except for the N394T and F463Y variants that failed to express at the oocyte surface. We observed that two variants (red bars, Figure 1) significantly differed from α3β4 control currents. These were the β4 R348C variant (corresponding to the human R349C) which showed significantly reduced currents (0.12 ± 0.43 normalized amplitude increase) in agreement with previous reports (Moriconi et al., 2011; Richards et al., 2011), and the D447Y variant (corresponding to the human D444Y) that showed significantly larger currents (3.13 ± 0.24 normalized amplitude increase). This rare SNP reported by Weiss and colleagues (Weiss et al., 2008) has not been previously characterized functionally. The 8 variants indicated in black bars (Figure 1) exhibited currents that did not differ significantly from control oocytes injected with α3β4wt (average current amplitude for a3b4 wt 0.88 ± 0.20μA) and were not further analyzed. The variants A90I (human T91I variant) and T374I (human T375I), showed slightly higher currents (blue bars in Figure 1) that were not significantly different from wt. However, since these 2 variants are the only SNPs in CHNRB4 that have been associated with nicotine dependence (Haller et al., 2012), we sought to further characterize them in neurons. Based on these results and criteria we selected the variants A90I, R348C, T374I, and D447Y of the β4 subunit for further analysis.

### β4 missense variants maintain the β4-rate-limiting property

We previously showed that β4 is rate-limiting for α3β4 activity as it has the capability to increase the density of α3β4 receptors at the plasma membrane, as well as to potentiate α3β4 currents when overexpressed *in vitro* and in a transgenic mouse model (Frahm et al., 2011). The ability of β4 to enhance nicotine-evoked currents depends on a unique single residue S435, which does not exist in other βnAChR subunits and can confer this capability to β2 when introduced in the corresponding arginine residue (Frahm et al., 2011).

To evaluate whether this unique property of β4 was conserved in the four selected β4 point mutants we next tested these point variants in overexpression conditions. Oocytes were injected with equal amounts of α3 and β4 transcripts (1:1 ratio) and compared to oocytes injected with 10 times more of the β4 transcript than of α3 (1:10 ratio). Representative traces of these recordings are shown in Figure 2A. It is interesting to note that the β4 R348C mutant not only leads to reduced agonist-induced currents, but also has slower kinetics of decay during ligand application (Figure 2A), consistent with reported electrophysiological recordings in cells (Moriconi et al., 2011). Upon overexpression, as previously observed for the native β4 (Frahm et al., 2011), nicotine-evoked currents increased 6.54 ± 0.43 fold for native β4, 6.12 ± 0.33 fold for β4A90I, 5.85 ± 0.68 fold for β4T374I, and 6.85 ± 0.07 fold for β4D447Y (Figure 2B). The β4R348C variant, which had lower currents at the 1:1 ratio (0.30 ± 0.030 fold amplitude normalized to α3β4wt, also showed increased currents reaching 2.97 ± 0.30 fold increase upon 1:10 overexpression (Figure 2B).

### Lentiviral constructs for targeted expression of α3β4 nAChRs in neurons

**Figure 3:**
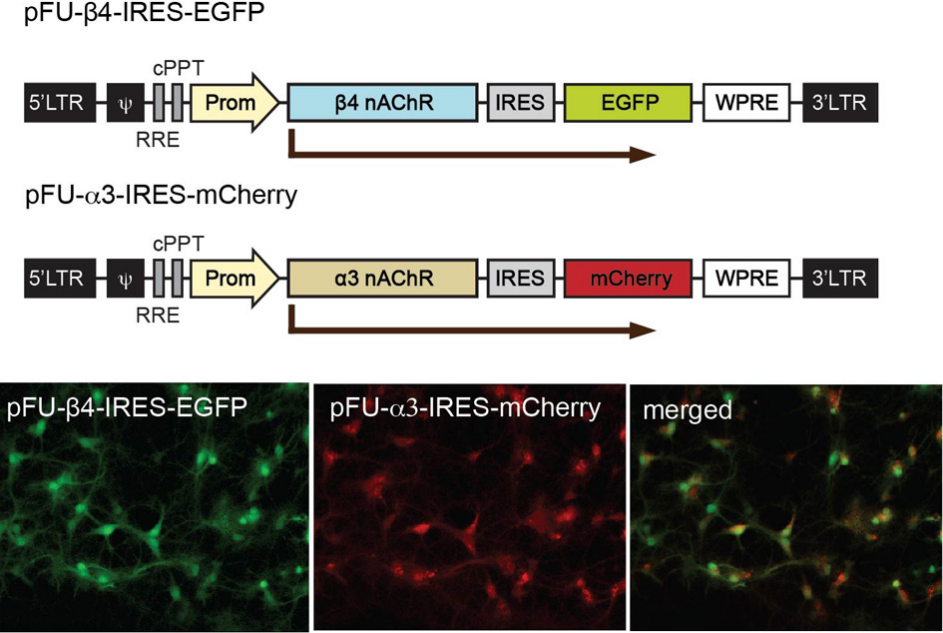
**Schematic representation of the viral constructs for expression of α3 and β4 nAChRs in neurons and for stereotactic injections in the Mhb.** Graphic representation of the lentiviral vectors encoding β4 (pFU-β4-IRES-EGFP) and α3 (pFU–α3-IRES-mCherry). The modules for viral expression are indicated: Ψ, packaging sequence; RRE, rev-responsive element; cPPT, central polypurine tract; Prom, human ubiquitin 2 promoter; IRES, internal ribosome entry site sequence; WPRE, woodchuck hepatitis B virus post-transcriptional regulatory element. Photomicrographs of direct fluorescence signal in rat hippocampal neurons transduced with the indicated β4 and α3 lentiviruses.

In order to analyze α3β4 nAChR currents in neurons and in mice, and evaluate the influence of the β4 missense variants, we constructed lentiviral vectors. To avoid potential problems with trafficking or assembly, we designed bicistronic constructs instead of generating fluorescent fusion proteins. The pFU-β4-IRES-EGFP construct contains the mouse β4 subunit (native or variant) followed by an internal ribosome entry site (IRES) and EGFP to monitor the level of infection, while pFU-α3-IRES-mCherry was designed to express the mouse α3 subunit and mCherry (Figure 3). As a control, we used pFU-PE, a lentivirus encoding the transmembrane domain of the PDGF receptor fused to EGFP (PE) (Auer et al., 2010). Prior to functional assays, the infection capabilities and reporter gene expression of the generated lentiviral particles were assayed in cultured rat hippocampal neurons. Bright direct fluorescence was observed in the soma and dendrites of neurons transduced with the α3 and β4 viruses (Figure 3).

### Electrophysiological recordings of β4 missense variants in neurons

**Figure 4:**
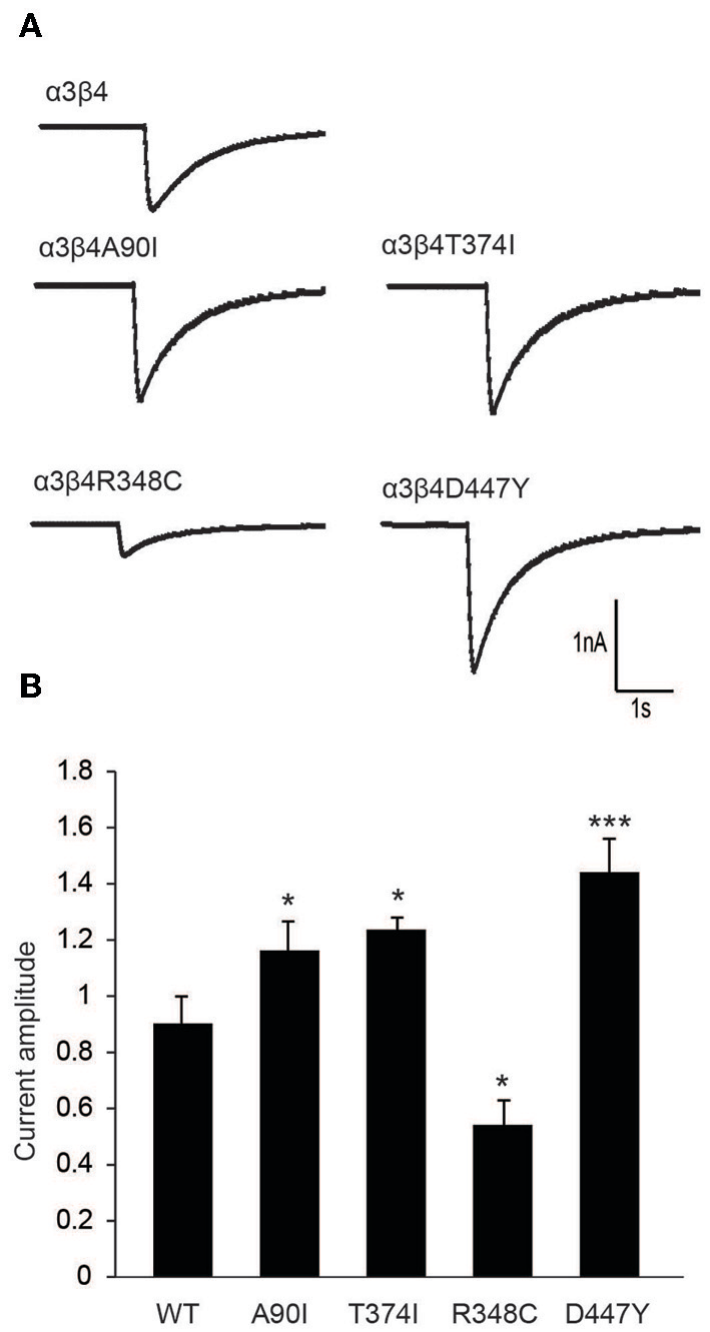
**Electrophysiological characterization of β4 variants in primary rat hippocampal neurons. (A)** Representative traces of nicotine-elicited currents in primary hippocampal neurons transduced with α3- and β4-carrying lentivirus **(B)** Quantification of nicotine-evoked currents show increased response when neurons were transduced with A90I, T374I, or D447Y β4 variants, while neurons transduced with point mutation R348C resulted in decreased response to nicotine (*n* = 15–22 neurons per condition). All values are expressed as mean ± s.e.m. One-Way ANOVA *F*_(4, 79)_ = 14.58, *p* < 0.0001; Dunnett's Multiple Comparison *post-hoc* ^*^p < 0.05, ^***^p < 0.001.

To further evaluate the modulatory effect of β4 variants on the response of neurons to nicotine, we performed patch clamp analyses in cultured rat hippocampal neurons co-transduced with lentivirus for α3 and β4 or β4 variants. Representative α3β4 nAChR currents recorded in co-transduced neurons upon pressure application of 30 μM nicotine are shown in Figure 4A. Quantitation of average current amplitudes revealed that the β4 point mutants A90I, T374I, and D447Y exhibited significantly larger responses to nicotine and that the R348C variant led to reduced nicotine-evoked current amplitudes, when compared to native α3β4 responses (Figure 4B). These results confirmed our electrophysiological studies in oocytes (Figure 1) and whole-cell recordings in transfected cells (Moriconi et al., 2011; Haller et al., 2012).

### *in vivo* lentiviral overexpression of β4 reconstitutes increased α3β4^*^ in the MHb of mice

**Figure 5:**
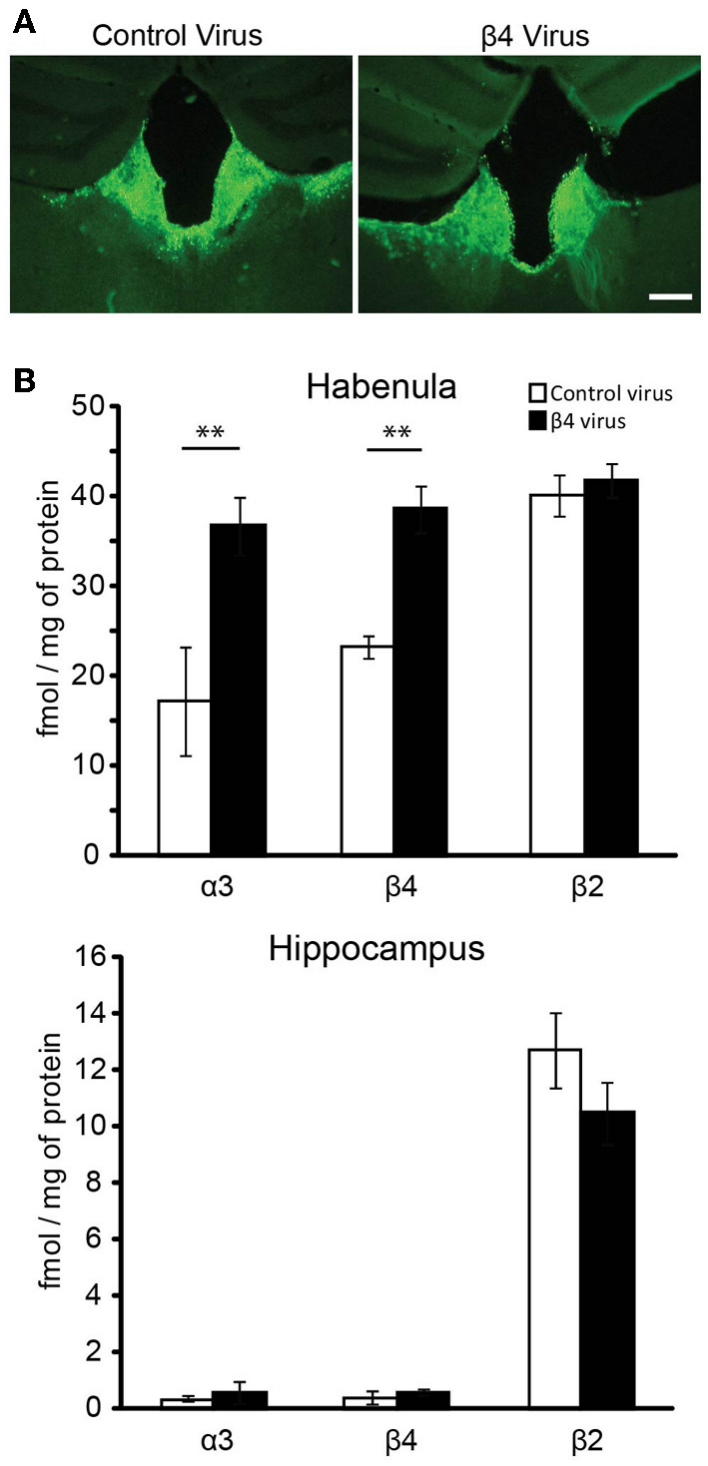
**Lentiviral mediated expression of β4 in the Mhb and quantification by immunoprecipitation. (A)** Representative coronal brain sections of mice injected with pFU-PE control virus or with the β4 containing virus pFU-β4-IRES-EGFP. **(B)** Quantitative immunoprecipitation, expressed as fmol/mg of protein of the solubilized [^125^I]epibatidine-labeled receptors immunoprecipitated by the anti-α3, -β4, or -β2 subunit specific antibodies (Grady et al., 2009). The solubilized [^125^I]epibatidine-labeled receptors were obtained from the habenula and hippocampus of C57BL/6 mice injected with either control virus or virus carrying the β4 nAChR subunit (*n* = 10 mice per group). ^**^p < 0.01, Student's *t*-test.

We have recently shown that transgenic Tabac mice expressing elevated levels of *Chrnb4* at endogenous sites, including the MHb, had increased cytisine resistant-^125^I-epibatadine ligand binding sites and increased α3β4^*^ nAChR currents, demonstrating that β4 has the ability to upregulate the number of α3β4^*^ receptors (Frahm et al., 2011). We wanted to assess whether selective overexpression of β4 only in the MHb would be able to reproduce this α3β4 upregulation *in vivo*. For this reason mice were sterotactically injected bilaterally in the MHb with either the β4-containing lentivirus or the pFU-PE control virus (Figure 5A). Quantification of α3, β4, and β2 subunits was performed by immunoprecipitation assays from 2% Triton X-100 habenula extracts obtained after solubilization of the membranes obtained from the habenula region of injected mice (Figure 5B). The hippocampus was used as an uninfected control region (Figure 5C). The indicated α3, β4, and β2 values correspond to the femtomoles of ^125^I-epibatidine labeled receptors immunoprecipitated per mg of protein. As shown in Figure 5B, there is a very pronounced increase in α3 and β4-containing receptors, but no change in the level of β2 receptors, in the habenula of mice injected with the β4 lentivirus. This suggests that, in the habenula, the β2 subunit is not coassembled in receptors containing the β4 subunit. The immunoprecipitations from hippocampus of mice injected in the MHb clearly indicate that there is no change in α3β4-containing and in β2-containing receptors, as expected from the restricted lentiviral-mediated expression of β4 by stereotactic injection. These results demonstrate that β4, as the rate-limiting subunit for assembly, can increase α3 incorporation and subsequent surface expression.

### Lentiviral-mediated expression of missense β4 variants in the MHb alters nicotine consumption in mice

**Figure 6:**
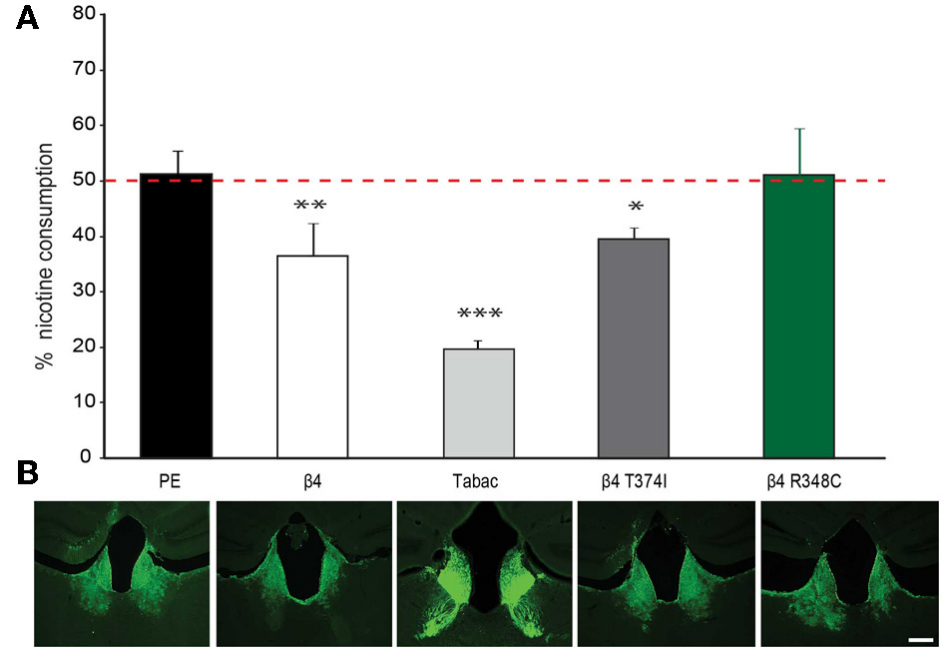
**Nicotine intake in mice injected in the Mhb with β4 and β4 missense variants. (A)** Two-bottle choice nicotine consumption in Tabac mice and in C57BL/6 mice after stereotactic, bilateral MHb injection of lentivirus carrying control (PE), wild-type β4 or β4 containing the R348C or T374I mutations constructs (*n* = 6–8 mice per group). All values are expressed as mean ± s.e.m. One-Way ANOVA *F*_(4, 29)_ = 25.25, *p* < 0.0001; Dunnett's Multiple Comparison *post-hoc* ^*^p < 0.05, ^**^p < 0.01, ^***^p < 0.001. **(B)** Representative images of coronal brain sections showing the site of lentivirus injection in MHb for PE, β4T374I, and β4R348C in C57BL/6 mice or eGFP expression in the MHb and fasciculus retroflexus in Tabac transgenic mice.

Given that lentiviral expression of only β4 is sufficient to upregulate α3β4 receptors *in vivo* in the habenula (Figure 5), and that transgenic Tabac mice expressing elevated levels of *Chrnb4* at endogenous sites, including the MHb, consume less nicotine (Frahm et al., 2011), we wanted to confirm the critical role of the MHb in nicotine consumption by employing lentiviral-mediated transduction to overexpress β4 in this particular brain region. We measured nicotine consumption using the two-bottle drinking test. Because nicotine solutions have bitter taste, control experiments were performed with a bitter solution (quinine). These measurements did not show significant differences in consumption between wt and Tabac mice (Frahm et al., 2011). We determined the relative volume of drank nicotine (diluted in 2% saccharin) compared to 2% saccharine sweetened water in wt, Tabac mice and wt mice injected with either a control virus, or virus encoding β4 or the β4 variants T374I and R348C. As shown in Figure 6, lentiviral-mediated overexpression of β4 in the MHb of injected wild-type mice is sufficient to cause a strong aversion to 50 μg/ml nicotine in a two-bottle choice paradigm (β4 injected mice: 36.58± 5.8%, *p* < 0.05) compared to mice injected with the PE control virus (51.35 ± 4.2%, *p* < 0.05). The nicotine aversion observed in wt mice injected with β4 lentivirus is less pronounced than the one observed in transgenic Tabac mice (11.86 ± 2.90% nicotine, *p* < 0.05), most likely because of higher expression of β4 achieved in Tabac transgenic mice [which contain more than 20 copies of the BAC encoding Chrnb4-a3-EGFP, (Frahm et al., 2011)] than in mice injected with the β4-lentivirus (Figure 6B). Mice injected with the lentivirus encoding the β4T374I gain-of-function variant also showed reduced nicotine intake (39.54 ± 3.6%, *p* < 0.05), while mice expressing the loss-of-function β4R349C variant showed no alteration in nicotine consumption (51.15 ± 8.4%, *p* < 0.05) with respect to the mice injected with the PE control virus (Figure 6B). Thus, our studies support a protective nature for the β4T374I variant in the animal model, in agreement with genome-wide association studies that linked the corresponding SNP T375I in *CHRNB4* to decreased risk for nicotine addiction in humans (Haller et al., 2012).

## Discussion

Chronic exposure to nicotine, the highly addictive component of tobacco, is thought to alter the neural circuits to promote changes that sustain the use of cigarettes (Paolini and De Biasi, 2011). The *CHRNA5-A3-B4* gene cluster encoding the α5, α3, and β4 and subunits of the nAChR has been linked with heavy tobacco use and a high risk of relapse (Saccone et al., 2009). The studies presented here establish that the β4 nicotinic receptor subunit regulates nicotine intake in mice, that restricted expression of β4 in the MHb is sufficient to produce nicotine aversion, and that four residues corresponding to *CHRNB4* SNP variants found in humans, A90I, T375I, R349C, and D444Y, have profound effects on nicotine currents and nicotine consumption in mice.

Three main conclusions can be drawn from these studies. First, β4 is rate-limiting in the formation of α3β4^*^ nAChRs. Hence when β4 is overexpressed, either in transgenic Tabac mice, carrying several copies of a transgene of the *Chrnb4-Chrna3-GFP-Chrna5* gene cluster, or by lentivirus-mediated transduction, the presence of supplementary β4 subunits favors the recruitment of α3 into additional α3β4 receptors. This β4-mediated upregulation of α3β4 nAChRs detected by ligand binding, electrophysiological and immunoprecipitation assays indicates that these additional α3β4 receptors are at the plasma membrane. This is important to consider in the context of nAChR dynamics and trafficking and the effect of nicotine on receptor upregulation. For instance, it has been shown that nicotine enhances pentamer assembly of the α3β4 nAChR combination (Mazzo et al., 2013). Since unassembled subunits are rapidly degraded by the endoplasmic-reticulum-associated protein degradation process (Christianson and Green, 2004), and efficient assembly of this nAChR subtype has been reported (Wang et al., 1998; Sallette et al., 2004), it is very plausible that increasing the number of available β4 subunits increases assembly and contributes to the α3β4 receptor upregulation that we have observed in Tabac mice and β4-lentivirus injected mice. Consistent with this hypothesis, recent studies have shown that α3β4 pentamers with three instead of two β4 subunits are less prone to degradation. This β4 protective effect depends on the endoplasmic reticulum export motif present in β4 _345_(LFM)_347_ which is lacking in α3 (Mazzo et al., 2013). Interestingly, this motif lies at one residue distance from the loss-of-function R349C variant that we have identified in our functional assays. Consistently, this mutant shows decreased currents and reduced membrane insertion (Moriconi et al., 2011; Richards et al., 2011). Hence these studies on β4 variants, together with our previous work showing that the native β4S435 residue has the unique capability to confer the rate limiting capability of β4 to other beta nAChR subunits, strengthens the view that the β4 subunit has a distinctive role in nAChR trafficking and assembly. The fact that this subunit has recently been linked to nicotine dependence further validates its key role in mediating nicotine responses.

Second, this study provides a novel paradigm to test the influence of β4 variants in nicotine consumption *in vivo*. Because of the unique capability of β4 to upregulate α3β4 nAChR activity, and because the MHb is such a small brain structure highly enriched in α3β4 receptors, the possibility to test the behavioral outcome of rare missense β4 variants in this system is valuable. The two SNPs analyzed here, A90I in the extracellular domain and T374I in the intracellular loop, have been linked to a reduced risk of nicotine dependence in GWAS and shown to increase nicotine currents in transfected HEK293 cells (Haller et al., 2012). Consistently, our screen of β4 missense variants identified these two SNPs, and an additional unstudied SNP, D447Y, as the variants with significantly higher nicotine-elicited currents in oocytes and in mammalian neurons. Subsequent viral-mediated expression of one of these gain-of-function alleles in the MHb resulted in strong aversion for nicotine, in support of the decreased risk of developing nicotine dependence in individuals carrying this allele. We also identified a variant with 70% less currents than native β4. This missense mutant, β4R348C, has not yet been identified in GWAS of nicotine dependence, but has been linked to sALS (Sabatelli et al., 2009; Moriconi et al., 2011). Cellular studies have shown that the β4R349C mutation, independent of the companion α subunit, causes a reduction in potency of both ACh and nicotine, decreases the density of whole-cell current evoked by maximal transmitter concentrations, and alters the kinetics of ACh-evoked whole-cell currents (Moriconi et al., 2011; Richards et al., 2011). Interestingly overexpression of this loss-of-function mutant in the habenula fails to induce nicotine aversion in mice in comparison to mice injected with native β4 or gain-of-function β4 variants. It is important to note that wild-type β4 and the β4348C mutant contain their native S435 residue, which is able to increase currents upon overexpression (Figure 2). Hence lentiviral overexpression of β4348C will produce more functional α3β4 receptors and subsequently higher nicotine mediated currents than if this mutant would be expressed in a knock-in situation, comparable to a β4R348C human carrier. Of course, the use of viral vectors has some limitations. Most notable are the facts that viral transduction results in overexpression of the carried gene, and that expression can sometimes occur in adjacent brain areas. While these points need to be considered, the facts that we have observed phenotypes consistent with the properties of the mutant receptors documented in oocytes and hippocampal neurons, and that very little expression of nAChR alpha subunits is detected in the surrounding cell types (Allen Brain Atlas images in supplementary material) that can combine with β4 to form functional receptors, support the conclusions drawn based on our viral expression studies. Another consideration to acknowledge is that the two-bottle free-choice drinking test paradigm used here measures aversion but not preference, and that mice injected with the non-β4 carrying control virus consumed a similar nicotine volume as mice overexpressing the β4R348C. Based on these considerations we can infer that if one single allele of this mutant was present, mice would have shown preference for nicotine. Thus, it would be interesting to assess whether sALS patients carrying this mutation are more prone to develop smoking dependence. From these data we can conclude that increased β4-mediated nAChR currents increase aversion to nicotine, while decreased currents decrease aversion, in favor of the hypothesis that the MHb-IPN pathway limits consumption of high doses of nicotine. Hence these results are relevant in the context of MHb-mediated control of nicotine consumption and the possibility to employ β4 to enhance or depress habenular activity.

Third, it is important to consider the fact that missense variants of β4 are so rare in the human population. For instance, the β4R348C loss-of-function variant that decreases α3β4^*^ currents to a similar extent as the α5D398N variant, and could potentially be linked to nicotine dependence, is present only in 1.4% of the human population (Table 1), in stark contrast with the rs16969968 corresponding to α5D398N present in 28.9% of smokers (http://www.ncbi.nlm.nih.gov/projects/SNP/snp_ref.cgi?rs=16969968). Given that the β4R348C mutation has been associated with sALS and that β4 is present in ganglionic and motor neurons, it is tempting to hypothesize that mutations in β4 would be too damaging to be carried to the progeny. The fact that smoking may enhance sALS (Armon, 2009) may underlie the importance of α3β4^*^ activity in enhancing disease susceptibility, in particular in association with ambient causes such as cigarette smoking. Hence very few studies have found significant associations of *CHRNB4* itself with smoking phenotypes, perhaps because it is the rate-limiting subunit when assembling β4^*^ nAChRs, suggesting that mutations in this subunit are less tolerated and thus found less often in the population.

## Author contributions

Inés Ibañez-Tallon designed research; Marta A. Ślimak, Jessica L. Ables, Silke Frahm, Beatriz Antolin-Fontes, Milena Moretti, and Cecilia Gotti performed research; Cecilia Gotti and Inés Ibañez-Tallon analyzed data; Inés Ibañez-Tallon and Jessica L. Ables wrote the paper.

## Conflict of interest statement

The authors declare that the research was conducted in the absence of any commercial or financial relationships that could be construed as a potential conflict of interest.
