# Supplementary material for: Habenular expression of rare missense variants of the β4 nicotinic receptor subunit alters nicotine consumption
Source: Front Hum Neurosci. 2014 Jan 27;8:12. doi: 10.3389/fnhum.2014.00012 (PMC3902282; doi:10.3389/fnhum.2014.00012)
Supplement: Supplementary file 2 [file Presentation1.PDF]

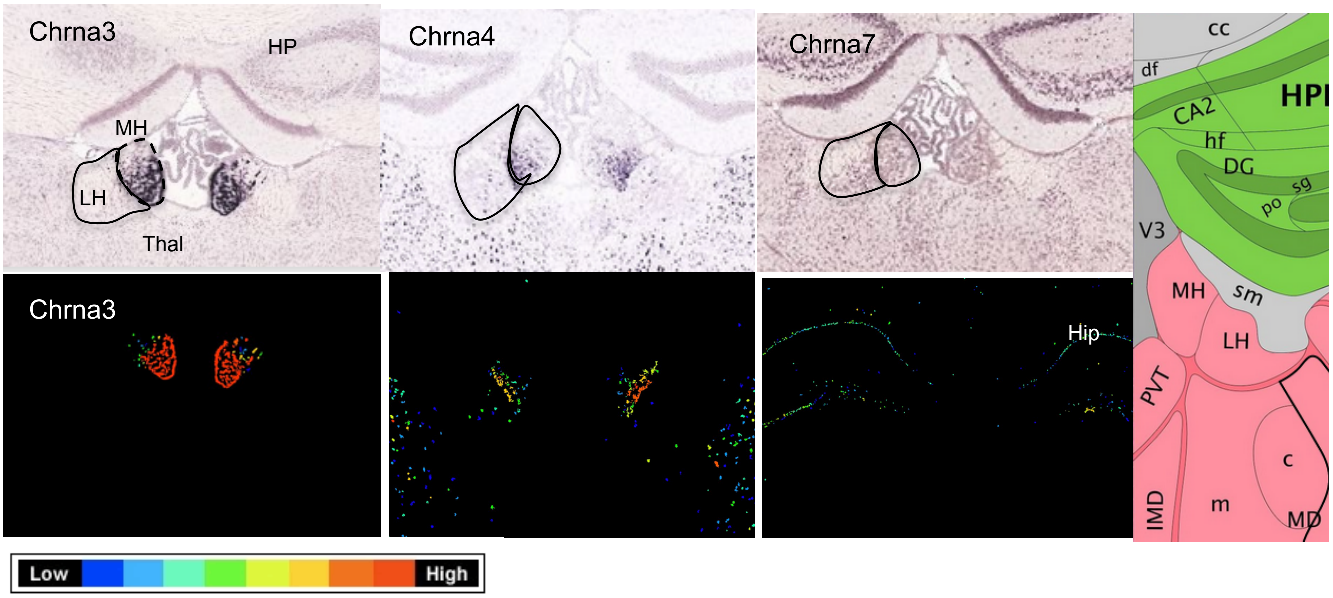

Allen Brain Atlas images showing in situ hybridization signal detected for Chrna3 (left) Chrna4 (center), and Chrna7 (right), and corresponding expression levels (low level in blue to high levels in red)

Chrna3 signal is detected only in the ventral medial habenula (MH). Chrna4 signal is present in the ventrolateral MH and in sparse cells in thalamus, and Chrna7 signal is absent in habenula and thalamus.
